# Development and characterization of serotype-specific monoclonal antibodies against the dengue virus-4 (DENV-4) non-structural protein (NS1)

**DOI:** 10.1186/s12985-018-0925-7

**Published:** 2018-02-06

**Authors:** Tesfaye Gelanew, Elizabeth Hunsperger

**Affiliations:** Dengue Branch, Division of Vector-Borne Diseases, National Center for Enteric and Zoonotic Infectious Diseases, Centers for Disease Control and Prevention (CDC), San Juan, PR USA

**Keywords:** Dengue virus 4, Non-structural protein 1, NS1, Diagnostic, flavivirus, Monoclonal antibody

## Abstract

**Background:**

Dengue, caused by one of the four serologically distinct dengue viruses (DENV-1 to − 4), is a mosquito-borne disease of serious global health significance. Reliable and cost-effective diagnostic tests, along with effective vaccines and vector-control strategies, are highly required to reduce dengue morbidity and mortality. Evaluation studies revealed that many commercially available NS1 antigen (Ag) tests have limited sensitivity to DENV-4 serotype compared to the other three serotypes. These studies indicated the need for development of new NS1 Ag detection test with improved sensitivity to DENV-4. An NS1 capture enzyme linked immunoassay (ELISA) specific to DENV-4 may improve the detection of DENV-4 cases worldwide. In addition, a serotype-specific NS1 Ag test identifies both DENV and the infecting serotype.

**Methods:**

In this study, we used a small-ubiquitin-like modifier (SUMO*) cloning vector to express a SUMO*-DENV-4 rNS1 fusion protein to develop NS1 DENV-4 specific monoclonal antibodies (MAbs). These newly developed MAbs were then optimized for use in an anti-NS1 DENV-4 capture ELISA. The serotype specificity and sensitivity of this ELISA was evaluated using (i) supernatants from DENV (1–4)-infected Vero cell cultures, (ii) rNS1s from all the four DENV (1–4) and, (iii) rNS1s of related flaviviruses (yellow fever virus; YFV and West Nile virus; WNV).

**Results:**

From the evaluation studies of the newly developed MAbs, we identified three DENV-4 specific anti-NS1 MAbs: 3H7A9, 8A6F2 and 6D4B10. Two of these MAbs were optimal for use in a DENV-4 serotype-specific NS1 capture ELISA: MAb 8A6F2 as the capture antibody and 6D4B10 as a detection antibody.

**Conclusion:**

This ELISA was sensitive and specific to DENV-4 with no cross-reactivity to other three DENV (1–3) serotypes and other heterologous flaviviruses. Taken together these data indicated that our MAbs are useful reagents for the development of DENV-4 immunodiagnostic tests.

**Electronic supplementary material:**

The online version of this article (doi: 10.1186/s12985-018-0925-7) contains supplementary material, which is available to authorized users.

## Background

Dengue is a serious disease of public importance with increasing worldwide spread. It is caused by an infection with any one of the four antigenically distinct dengue virus serotypes (DENV1–4). Current diagnosis of an acute DENV infection primarily relies on reverse transcription polymerase chain reaction (RT-PCR), a highly sophisticated test. To improve dengue case detection in dengue endemic countries, a highly sensitive, specific and simple rapid assay is needed to provide early support for patients and to accurately differentiate dengue from other acute febrile illnesses. DENV non-structural protein 1 (NS1) is a unique diagnostic marker for early detection of DENV compared to serological tests (*i.e.,* anti-DENV IgM) because it is detected in the serum of DENV-infected patients as early as one day post onset of symptoms (DPO) to 18 DPO at concentration up to 50 μg/ml and it is a confirmatory test [1]. Whereas anti-DENV IgM is not present until five days after illness onset, can cross react with other flaviviruses thus not considered a confirmatory test especially in regions where multiple flaviviruses co-circulate. NS1 is highly conserved glycoprotein and possesses both group-specific and serotype-specific epitopes hence it can discern DENV from other flaviviruses and has the potential to differentiate between DENV serotypes [2].

Rapid tests such as the NS1 enzyme-linked immunosorbent assay (ELISA) are commercially available for DENV with relatively good sensitivity and specificity [3]. Recently several studies have critically evaluated the performance of the current commercial NS1 ELISA kits by DENV serotype. Results from these studies showed that these tests had lower sensitivity for the detection of DENV-4. Additionally, these commercial tests had decreased sensitivity in detecting secondary dengue infections, common in dengue endemic countries [3–9]. Commercially available NS1 antigen (Ag) tests manufactured by BioRad (Platelia) and Panbio (Dengue Early) both have low sensitivity to DENV-4 infections [8, 10–12]. Previous evaluations of Dengue Early test showed a sensitivity as low as 19% for DENV4 [6, 8, 13–15]. Although the overall sensitivity of Platelia test was good however when comparing the sensitivities between all DENV serotypes, the BioRad test had the lowest sensitivity for DENV-4 (58.3%) [10].

Our rationale to develop a DENV4 ELISA was due to the limited sensitivity of NS1 tests for detection DENV-4 infections. Recent evaluation study using retrospective samples from South America showed lower sensitivity of seven commercially available NS1 Ag tests for DENV-4 compared to the other three serotypes [7]. Also, meta-analysis for DENV detection in Asia showed the lowest sensitivity of the commercial NS1 Ag tests was against DENV-4 detection [16]. Further, studies conducted in Brazil showed the lower sensitivity of Platelia NS1 Ag ELISA (BioRad) for DENV4 detection [13, 14]. Collectively, these data suggest the need for development of new NS1 Ag detection test that incorporates monoclonal antibodies (MAbs) with higher sensitivity to DENV-4.

In order to improve the performance of NS1 Ag detection tests with higher sensitivity to DENV-4, it is important to understand why the current NS1 Ag detection tests failed to detect DENV4 infections efficiently. Almost all the present commercial NS1 Ag detection tests are based on cross-reactive anti-NS1 MAbs to all four DENV serotypes due to common epitopes. Epitope mapping studies have identified epitopes LX1 (113-YSWKTWG-119) and LD2 (24-VHTWTEOYK-32) that are common to all four DENV serotype [2, 17, 18]. Several studies showed the absence of significant amino acid sequence variation in the epitopes of serotype-cross-reactive MAbs [2, 19, 20]. This is consistent with Aryati and colleagues observation that there was no link between NS1 gene (amino acid) sequence variation and the poor performance of Dengue Early (Panbio) for DENV-4 detection [6]. Factors other than NS1 gene (and/or amino acid) sequence variation may impact the bio-accessibility and/or binding of the conserved epitopes to MAbs depending on the serotype. For example, the bio-accessibility of the conserved epitopes may vary according to the serotype/genotype when the NS1 protein is folded and assembled to form the NS1 hexamer. Hence, this suggest the common linear epitopes targeted by MAbs could be partially accessible on the NS1 hexamer isoform of DENV-4. The crystal structure by Akey and colleagues identified 108 linear epitopes that were mapped to the hexamer form of the protein primarily to the wing domain, C-terminal tip of the Beta bladder and Beta roll which are the most exposed epitopes. This study also demonstrated that the Beta rolls are most likely unexposed epitopes and the spaghetti loop and glycosylation sites are exposed epitopes [21]. Future studies may be able to use the crystal structure predicted exposed epitopes to design novel NS1 immunogenic MAbs.

To overcome the current limitations of the MAbs for NS1 tests, we used a small-ubiquitin-like modifier (SUMO)*- DENV-4 rNS1 fusion protein to develop new anti-NS1 MAbs specific to DENV-4. Due to the external hydrophilic surface and inner hydrophobic core properties of SUMO, fusion of NS1 to SUMO is an efficient method of expressing this membrane bound protein allowing for improved NS1 solubility and proper folding in order to develop DENV4 specific MAbs. Using a pair of these antibodies, we developed a DENV-4 serotype-specific NS1 capture ELISA. The serotype specificity and sensitivity of this ELISA was evaluated using (i) supernatants from four DENV (1–4)-infected cell cultures, (ii) rNS1s from all the four DENV (1–4), and (iii) rNS1s of related flaviviruses (yellow fever virus; YFV and West Nile Virus; WNV).

## Methods

### Preparation of NS1 antigen from DENV-infected Vero cells

DENV infected Vero cell culture supernatant from each of the four DENV serotypes was produced as previously described [20] with slight modifications. Briefly, Vero cells (ATCC, VA, USA) were infected at multiplicity of infection (MOI) =0.1 and grown in M199 medium (Gibco/Life Technologies, Grand Island, NY, USA) supplemented with 5% super low IgG FBS (Invitrogen, Carlsbad, CA, USA) and 1% Gentamicin (Gibco), 1% Penicillin–Streptomycin (Gibco), 3% sodium bicarbonate (Gibco). In order to avoid cell lysis and thereby the release of proteolytic enzymes into the culture medium, DENV-infected Vero cells were monitored every day for cytopathic effect (CPE), and culture supernatants were harvested and filtered on day 3 or 4 post infection at ~ 50% CPE. The same procedure was followed for control antigen from mock-infected Vero cells. Culture medium was concentrated (3X) by using Amicon, Centricon-30 K (Millipore, Billerica, MA, USA), and treated with SIGMAFAST protease inhibitor cocktail (Sigma, St. Louis, MO, USA). The presence of secreted native NS1 in these culture supernatants was confirmed using a serotype-cross-reactive Dengue Early NS1 ELISA (Panbio Diagnostics, Brisbane, Australia). Thereafter, these culture supernatants were stored at −80°C until use.

### Recombinant antigens

All recombinant (r) flavivirus NS1 antigens used in this study were purchased from NativeAntigen (Oxfordshire, UK). Recombinant sumo protein was purchased from LifeSensors (Malvern, PA, USA).

### Cloning DENV-4 NS1 into pI-secSUMO* vector

Total viral RNA was isolated from viral seeds of DENV4 (H421, a prototype strain) infected C6/36 cells (*Aedes albopictus* cell line, ATCC) using the QIAamp Viral RNA Mini kit (QIAGEN, Gaithersburg, MD, USA), and a complimentary DNA (cDNA) of DENV-4 NS1 gene was reverse transcribed using Super Script III one step reverse transcription polymerase chain reaction (RT PCR) kit (Invitrogen, Carlsbad, CA, USA) and paired primers (forward: 5’-ATACGTCTCTAGGTGACACGGGTTGTGCGGTG-3′) and (reverse; 5’-GCGTCTAGATTAGGCCGATACCTGTGATTT-3), according to manufacturer’s instructions. These primers were designed to introduce restriction sites for *BmsB1* and *Xbal* in order to clone the full length of DENV-4 NS1 gene into a directional pI-secSUMO* cloning vector (LifeSensors, Malvern, PA, USA). Prior to primer design, we checked for the complete absence of *BmsB1* and *Xbal* restriction sites in the full-length of DENV4 NS1 gene sequence using a NEBcutter V2.0 (http://nc2.neb.com/NEBcutter2/).

The underlined nucleotide sequences in the forward and reverse primers represent the actual first codons, and the complementary sequence to the last codons of the DENV4 NS1 gene, respectively. In 5′ end of the forward primer (ATA) was added to enhance the efficacy of PCR product end digestion by *BmsB1.* The sequence, CGTCTCT, was added as a *BmsB1* restriction cleavage site for insertion of NS1 into the multiple cloning sites of the pI-secSUMO* vector*,* and GGT represented the last codon of the SUMO* tag. In the 5′ end of the reverse primer, GCG was added to enhance the efficacy of *Xbal* restriction digest at the PCR product end, TCTAGA was added to serve as an *Xbal* restriction cleavage site for insertion of NS1 into the multiple cloning sites of the pI-secSUMO* vector, and TTA represented a reverse complement of the stop codon.

The amplicons generated were visualized by gel electrophoresis using a 1% low melting point agarose (LMPA) (Invitrogen), and the appropriate band was excised, and purified using a QIAGEN Gel Extraction Kit (QIAGEN, Gaithersburg, MD, USA) and double-restriction enzyme digested with *BmsB1* and *Xbal* (New England BioLabs, MA, USA). Since the optimal temperatures and buffers for these two restriction enzymes are different, a two-step restrictions digest was performed. First, amplicons were digested with *Xbal* at 37 °C for 1 h, the solution was then centrifuged at 10,000 g for 30 min and supernatant was discarded. The DNA pellet obtained was washed twice with an ice cold 70% ethanol, air dried, and re-suspended in Milli-Q water. The re-suspended DNA pellet was digested with *BsmB1* for 1 h at 55 °C. Then the pI-secSUMO* plasmids were linearized with *BsmB1* in order to generate two overhang ends which are complementary to the double-digested PCR product ends. Both the double-digested PCR products and linearized plasmids were then visualized on a 2% LMPA gel; the appropriate bands were excised, purified, and ligated overnight at 16°C using T4 DNA Ligase (Invitrogen) with 3:1 ratio of double-restricted amplicons (NS1 gene) to linearized pI-secSUMO* plasmids. The ligated solution was then immediately transformed into chemically competent DH5α *E. coli* cells (Invitrogen) as per transformation protocol described elsewhere [22]. Positive DH5α *E. coli* clones with recombinant SUMO*-NS1 gene were then selected by colony PCR using forward primer polH (described below) and the reverse primer. Additionally, to verify the integrity of NS1 gene, recombinant plasmid DNAs isolated from PCR-positive clones using QIAGEN Plasmid MiniPrep Kit (QIAGEN, California, USA) were sequenced in both directions using two external primers (polH, 5’-GGATTATTCATACCGTCCCACCAT-3 and Tn7, 5’-CTGGGTGTA GCGTCGTAAGCTAATAC-3). Plasmid DNAs from pFastBac™ TOPO donor plasmid (Invitrogen) containing SUMO*-NS1 fusion construct were then transformed into chemically competent DH10bac *E. coli* cells (Invitrogen) according to the transformation protocol as previously described [22]*.* Positive clones were selected by blue and white screening assay followed by colony PCR. Then recombinant Bacmid DNA was isolated from positive clones using QIAGEN Plasmid DNA Miniprep kit (QIAGEN), and sequenced for integrity in both direction using primer pair polH and Tn7. The isolated Bacmid DNAs were stored at 4 °C until use.

### Expression of SUMO*-DENV-4 NS1 fusion protein in *Sf21* cells

**Fig. 1:**
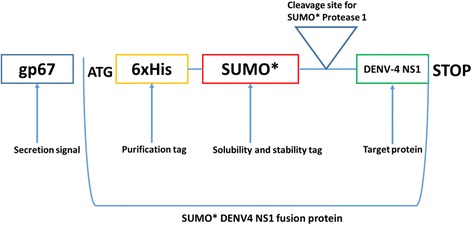
SUMO*-DENV-4 NS1 fusion gene construct. Schematic showing the DENV-4 fusion construct within the SUMO* cloning vector used to express recombinant protein in *Spodoptera frugiperda (Sf)21* cells

Expression of SUMO*-NS1 fusion protein was done in *Spodoptera frugiperda (Sf)21* cells (Invitrogen). *Sf21* cells were transfected using 3 μg of Bacmid DNA for high titer viral stock production, which was later used for infection of these cells at an MOI = 1. Since  pl-secSUMO* plasmid contains an upstream gp67 secretion signal of the SUMO* fusion which results in secretion of the SUMO*-rNS1 into the cell culture medium (Fig. 1), only culture supernatant of *Sf21* cells was harvested on day 3 post infection. The culture supernatants (containing soluble SUMO*-NS1 fusion protein with 6xHis-tag) was concentrated (5X) and then purified by immobilized metal affinity chromatograph (IMAC) under native conditions. The purity as well as the molecular size of the fusion protein was determined by Coomassie blue stained 12% SDS-PAGE gel (Invitrogen) under both reducing and non-reducing conditions, and confirmed by western blot assay. The proper folding of the SUMO* tagged DENV-4 rNS1 protein was confirmed by commercial serotype cross-reactive Dengue Early (Panbio), which comprises MAbs reactive to the native hexameric NS1 in serum samples of dengue patients.

### Western blot analysis

Fused protein of SUMO*-DENV-4 NS1 and unfused DENV-4 rNS1 protein at concentration of 2 μg or 100 ng were separated using Novex NuPAGE 12% SDS-PAGE gel (Life Technologies) under non-reduced, heat-denatured or non-denatured conditions. Protein size discrimination was determined with Molecular Weight (MW) standards (116, 66, 45, 31, 21 kDa) and MagicMark XP Protein standards (20–220 kDa, Invitrogen). For western blot analysis, the proteins were transferred to 0.22 μm nitrocellulose membranes (Invitrogen), blocked overnight at room temperature (RT) with 5% non-fat dried milk (NFDM) in phosphate buffered saline (PBS) with 0.05% Tween20 (NFDM-PBST). For SUMO*-DENV-4 NS1 protein analysis by western blot, the membrane was incubated with anti-HisTag. For the characterization of MAbs elicited from SUMO*-DENV-4 NS1, the nitrocellulose membranes were incubated with the primary MAbs that were produced against the fusion protein. Following incubation with primary antibody (generated MAbs), a secondary anti-mouse peroxidase detector antibody diluted at 1:10,000 (KPL, Gaithersburg, MD, USA) was used. The substrate SuperSignal West Pico solution (Pierce/Thermo Scientific) was used to detect proteins.

### Mice immunization, and isolation of anti-DENV-4 NS1 MAbs secreting hybridoma clones

The immunization and production of hybridoma clones were performed by Custom Antibody Generation Services (Celtein Biosciences, LLC; Monroe, Ohio) using our SUMO* DENV-4 NS1 fusion protein. To produce anti-NS1 MAb-secreting hybridoma clones, female BALB/c mice (6–8 weeks) were immunized with purified soluble SUMO*-DENV-4 NS1 fusion protein in complete Freud’s adjuvant followed by four boosts with incomplete Freund’s adjutant in 14 days of interval between boosts. Four days after the last boost, primed spleen cells were isolated aseptically from mice with the highest titer against SUMO*-DENV-4 NS1 fusion protein and unfused DENV-4 rNS1 (expressed in mammalian cells). These primed spleen cells were then fused with SP2/0 cells as previously described (Kohler and Milstein, 1975). To select desired MAb secreting hybridomas, culture supernatants from each clone was evaluated by indirect ELISA (iELISA) using both SUMO*-DENV-4 NS1 fusion protein and unfused DENV-4 rNS1 protein and fixed cell ELISA [20]. Further, hybridoma culture supernatants was also screened against recombinant SUMO protein by iELISA.

### MAb production, purification and characterization

MAb production from positive hybridoma clones (3H7A9, 6D4B10 and 8A6F2) was carried out using a CELLine 1000 bioreactor (BD Biosciences; Sparks, MD, USA) according to the manufacturer’s instructions and as per protocols described in our previous report [20]. Purification MAb was done using VivaPure Maxiprep Protein G Spin Columns (Satorius Stedim, Bohemia, NY, USA) as per manufacturer’s instructions. MAbs were characterized using fixed cell ELISA and iELISA as previously described [20]. For the iELISA, we coated 96-well microtiter ELISA plates with monomeric rNS1 at a concentration of 1 μg/ml in PBS overnight at 4 °C and then blocked with PBS (pH 7.4) containing 5% NFDM and 0.5% Tween-20 for 30 min at RT. Following blocking step, the microtiter plates were washed 3X with PBS + 0.05% Tween-20 (PBST) and treated with hybridoma supernatants in duplicate and diluted to 1:100, 1:500, 1:1000 and1:2000 in wash buffer and incubated for 1 h at 37 °C. After 5X washes, goat anti-mouse IgG horseradish peroxidase (HPR)-conjugated (KPL,Gaithersburg, MD) diluted to 1:1000 in blocking buffer was added and incubated for 1 h at 37 °C in a humidified chamber. This was followed by 5X washes and then the addition of 3,3_,5,5_-Tetramethylbenzidine (TMB) liquid substrate (KPL) and incubated for 15 min at RT in the dark. The reaction was stopped by TMB stop solution (KPL) and the OD was determined by ELISA ELx800 microplate reader (BioTech®, VT, USA) using a 450 nm filter. The fixed cell ELISA used DENV infected Vero cells that were seeded onto 96-well culture plates at a density 2 × 10^5^cells/well and incubated at 37 °C in 10% CO_2_ for 2 days to obtain a desirable 90% confluent monolayer. Cells were then infected with each of the four DENV serotypes at an MOI = 0.1. Five days post infection, the culture medium was removed and cells were fixed for 30 min at − 20 °C with equal volume of ethanol and methanol. The fixed cells were then washed 5X with PBS and blocked for 30 min at RT with 5% NFDM in PBST. MAbs were added to the plate at serial dilutions of 1:100, 1:500 and 1:1000 and incubated for 1 h at 37 °C. After 5X washes with PBS, HRP-conjugated goat anti-mouse IgG (KPL) diluted to 1:1000 in blocking buffer was added and incubated at 37 °C for 1 h in a humidified chamber. The plate was washed 5X with PBS, ABTS (2,2_-Azinobis[3-ethylbenzothiazoline-6-sulfonic acid]-diammonium salt) substrate (KPL) was added, incubated in the dark at RT and the OD was measured in ELx800 microplate reader using a 405 nm filter. All MAbs dilutions were tested in duplicate. Positive reactivity was determined if the average OD value for duplicate tests was greater than the corresponding average of mock-infected cells under the same conditions. MAbs isotypes were determined using a Mouse MAbs Isotyping kit (Pierce/ Thermo Scientific) according to the manufacturer’s instructions. Purified and dialyzed MAbs were biotinylated with a spacer arm biotin (NHS-PEG_4_-Biotin) using EZ-Link™ NHS-PEG4-Biotinylation Kit (Pierce/Thermo Scientific, Rockford, IL, USA).

### Epitope mapping by competition ELISA

In order to determine whether our MAbs recognized the same epitope or distinct epitopes on the DENV4 NS1, we performed a competition ELISA as previously described [20].

### Sequencing the antigen binding sites of MAbs

Sequencing the antigen binding sites (the variable regions of light (VL) and heavy (VH) chains) of the three MAbs were performed by customer service (GenScript, NJ, USA) in order to determine the identity of these MAbs. Five single colonies with the correct variable light and heavy chains genes inserted per each hybridoma clones were sequenced. After performing a multiple sequence alignments of the five peptide sequences using ClustalW2 (http://www.ebi.ac.uk), a consensus sequence for both V_L_ and V_H_ of each hybridoma clone was obtained. Finally, multiple alignment was performed among V_L_ consensus sequences and V_H_ consensus sequences to determine the identity of binding regions; the three complementarity determining regions (CDR1, CDR2, and CDR3) of MAbs.

### Development of DENV-4 specific NS1 capture ELISA

NS1 capture ELISA was developed by utilizing two of the MAbs as a capture and detector antibody. In order to determine the optimal capture/detector pair for detection of DENV-4 NS1 antigen, each MAbs was tested either as a capture or a detector antibody. The optimal concentrations of capture and detector antibodies were determined by checkerboard titration ELISA. Microtiter plates were coated with MAb 8A6F2 (100 μl/well) diluted in bicarbonate buffer (pH 9.6) at concentration 10 μg/mL and incubated overnight at 4 °C. Next day, excess unbound capture MAb was removed and thereafter microtiter plates were blocked with 200 μl/well of 5% NFDM or 2% bovine serum albumin (BSA) (*w*/*v*). After 45 min incubation, microtiter plates washed 3X with PBST and thereafter 100 μl/ml of rNS1 antigen (0.5 μg/ml) and serum sample diluted 1:1 in PBST was added and incubated at 37 °C for 60 min. Following incubation and 3X washes, 100 μl/well of biotinylated MAb 6D4B10 diluted at 1:2000 in PBST were added and incubated for 1 h at 37 °C. After 3X washes, 100 μl/well peroxidase-conjugated Streptavidin was added and then microtiter plates were incubated at 37 °C for 30–60 min. After 5X washes, the reaction was visualized by adding 100 μl/well TMB liquid substrate and incubating at RT in the dark for 15 min. The reaction was then stopped with 100 μl/well TMB stop solution. The optical density (OD) was measured at 450 nm on ELISA ELx800 microplate reader. Each sample was tested in triplicate.

### Testing culture supernatants from DENV (1–4) infected Vero cells and rNS1 of flaviviruses

We determined the serotype-specificity of the above described NS1 capture ELISA using culture supernatants obtained from DENV-infected Vero cells and commercially available rNS1 of flaviviruses, including all four DENV serotypes and expressed in mammalian cell line (NativeAntigen, Oxfordshire, UK). The presence of NS1 in those culture supernatants were pre-confirmed using a Dengue Early (Panbio) NS1 capture ELISA.

## Results

### SUMO*-DENV-4 NS1 fusion protein expressed was the correct conformation of DENV NS1

**Fig. 2:**
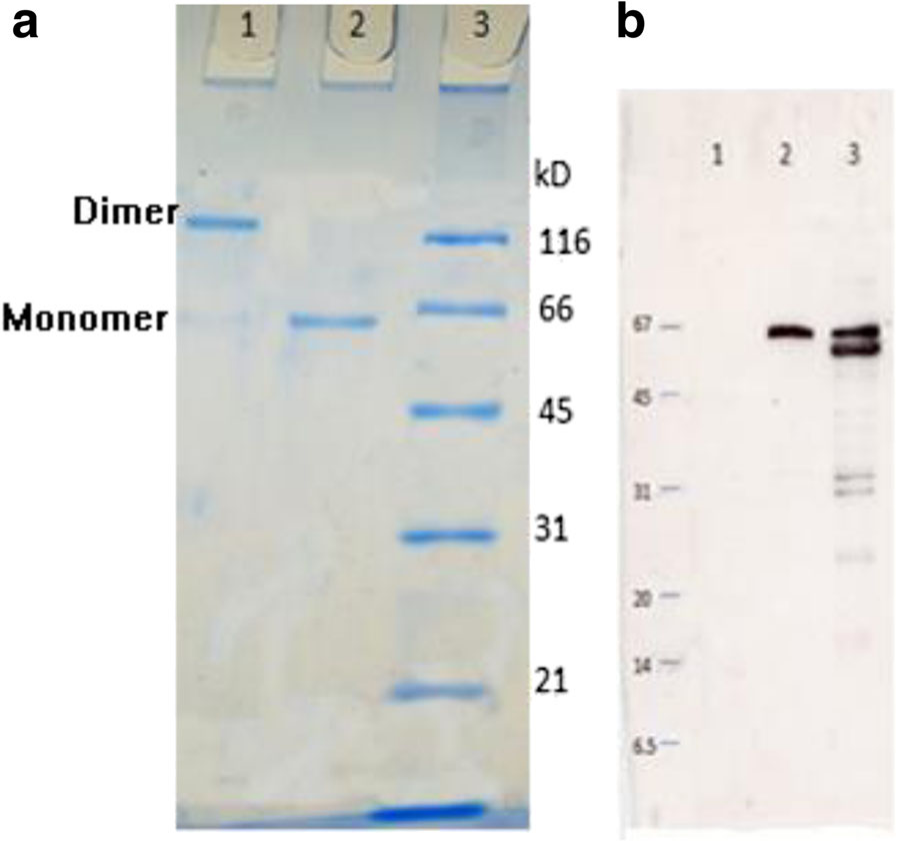
SUMO*-NS1 fusion protein expression analysis. **a** Coomassie-blue stained 12% SDS-PAGE gel. Lane 1: SUMO*-DENV-4 NS1 under non-reducing conditions (dimer); Lane 2: SUMO*-DENV-4 NS1 under reducing conditions (monomer); Lane 3: MW of markers in kDa. **b** Western blot of SUMO*-DENV-4 NS1 protein using anti-HIS antibody: Lane 1: Molecular Marker; Lane 2: secreted NS1; Lane 3: cell lysate of solubilized of SUMO*- DENV-4 NS1

Affinity purified SUMO*-DENV-4 NS1 fusion protein expressed in *Sf21* cells appeared as a monomer (~ 62 kDa) and a dimer (~ 120 kDa) in 12% SDS-PAGE under reduced and non-reduced conditions, respectively (Fig. 2A). The expected sizes of monomeric and dimeric native DENV4 NS1 expressed in mammalian cells are 48 kDa and 80 kDa, respectively [23]. Considering the molecular weight of the SUMO* tag, which is~11.5 kDa, the observed sizes on SDS-PAGE matched to the sizes of monomeric and dimeric DENV4 NS1, respectively. Also, appearance of a single band on 12% SDS-PAGE (Fig. 2B) confirmed the purity of the SUMO*-NS1 fusion protein. The correct conformation of the expressed and purified protein was confirmed by commercial cross-serotype reactive Dengue Early NS1 capture ELISA (Panbio). SUMO tag is cleavable from the target, DENV-4 rNS1 using SUMO protease without leaving no extraneous residues attached to the target protein. Despite this we used SUMO*-DENV rNS1 fusion protein for immunization of mice. Our strategy has an advantage over immunization with cleaved DENV-4 NS1 because it avoids the time and effort required to cleave the SUMO* tag from DENV4 NS1. In addition, it reduces the loss of protein during cleavage and re-purification steps to remove the SUMO* tag.

### Characteristics of DENV-4 monoclonal antibodies

**Fig. 3:**
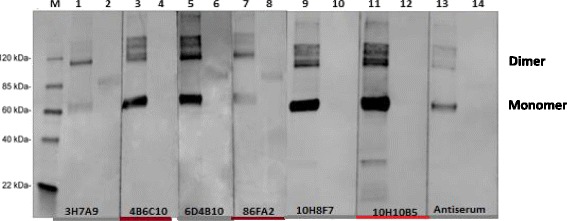
Anti-NS1 Monoclonal Antibodies (MAbs) reactivity to SUMO*-NS1 fusion protein. Western blot assay to determine the reactivity of hybridoma supernatants to SUMO*-NS1 fusion protein as well as unfused commercial rNS1 protein expressed in mammalian cell,; M; molecular markers; Lanes 1 and 2: 3H7A9; Lanes 3 and 4: 4B6C10; Lanes 5 and 6: 6D4B10; Lanes 7 and 8: 8A6F2; Lanes 9 and 10: 10H8F7; Lanes 11 and 12: 10H10B5; Lanes 13 and 14: Antiserum of mouse ME 1:100, reactivity to SUMO*-DENV NS1 fusion protein and unfused commercial DENV rNS1 expressed in mammalian cell, respectively. The sizes of the monomer and dimer for SUMO-rNS1 are ~ 62 kDa and 120 kDa due ~ 15 kDa SUMO tag whereas the sizes for monomer and dimer of unfused commercial rNS1 (NativeAntigen) are 48 kDa and 80 kDa

**Fig. 4:**
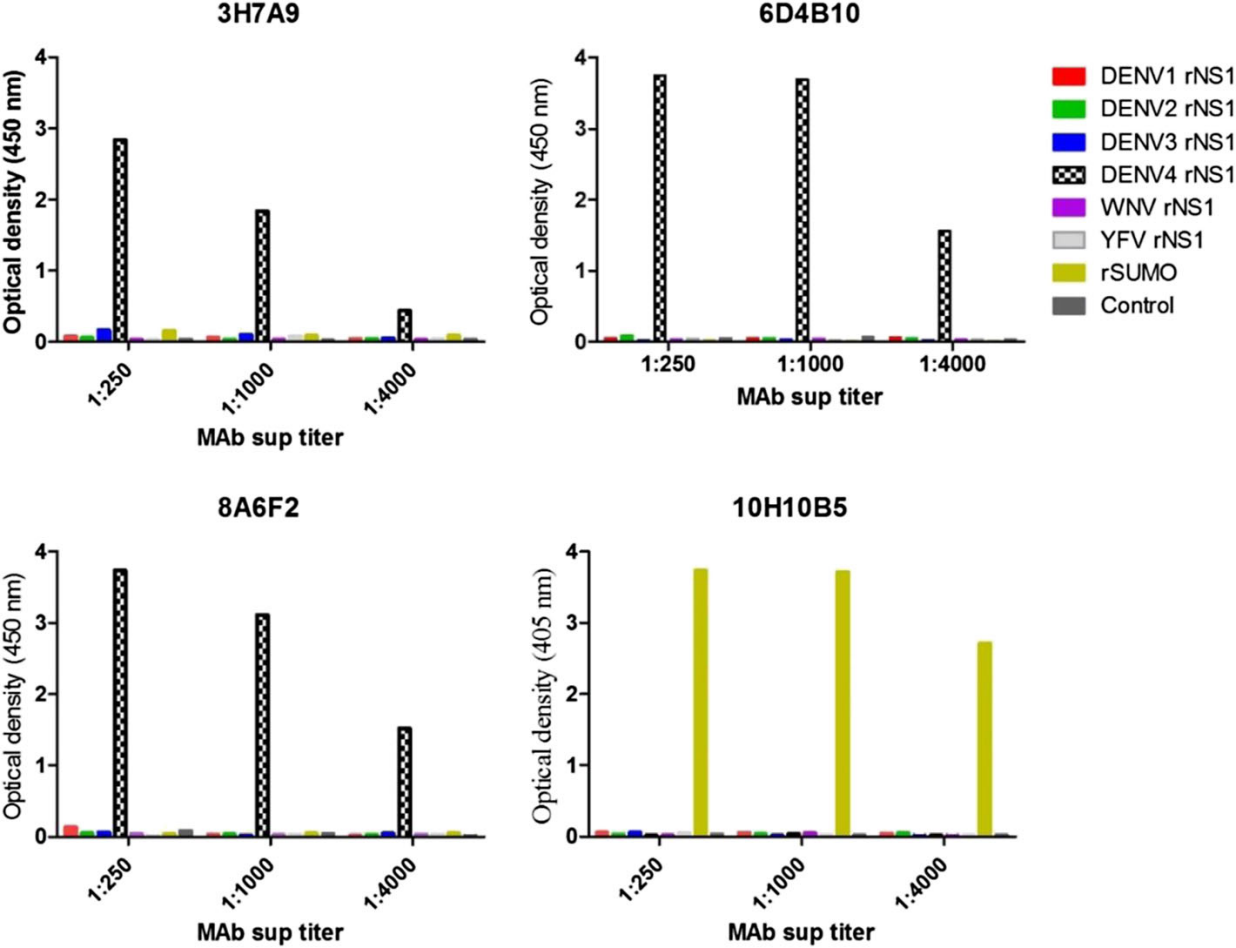
Serotype-specificity of monoclonal antibodies (MAbs 3H7A9, 8A6F2, 6D4B10 and 10H10B5) against DENV-4 determined by iELISA. The bars show mean optical density (OD) at 450 nm which measures MAb reactivity to NS1s to all four DENV-4 serotypes, yellow fever virus (YFV), West Nile virus (WNV) and SUMO protein. MAbs 3H7A9, 8A6F2, and 6D4B10 represent anti-NS1 MAbs specific to DENV-4. MAb 10H10B5, which is reactive to SUMO*, represents one of the three non-specific MAbs generated from immunization of SUMO*-DENV-4 rNS1 fusion protein. The OD value for DENV-4-serotype specific MAbs against rNS1 of other flaviviruses and recombinant SUMO protein was < 0. By contrary, MAbs reactive only to SUMO* protein (10H10B5) did not show any reactivity to rNS1 to YFV and WNV

**Fig. 5:**
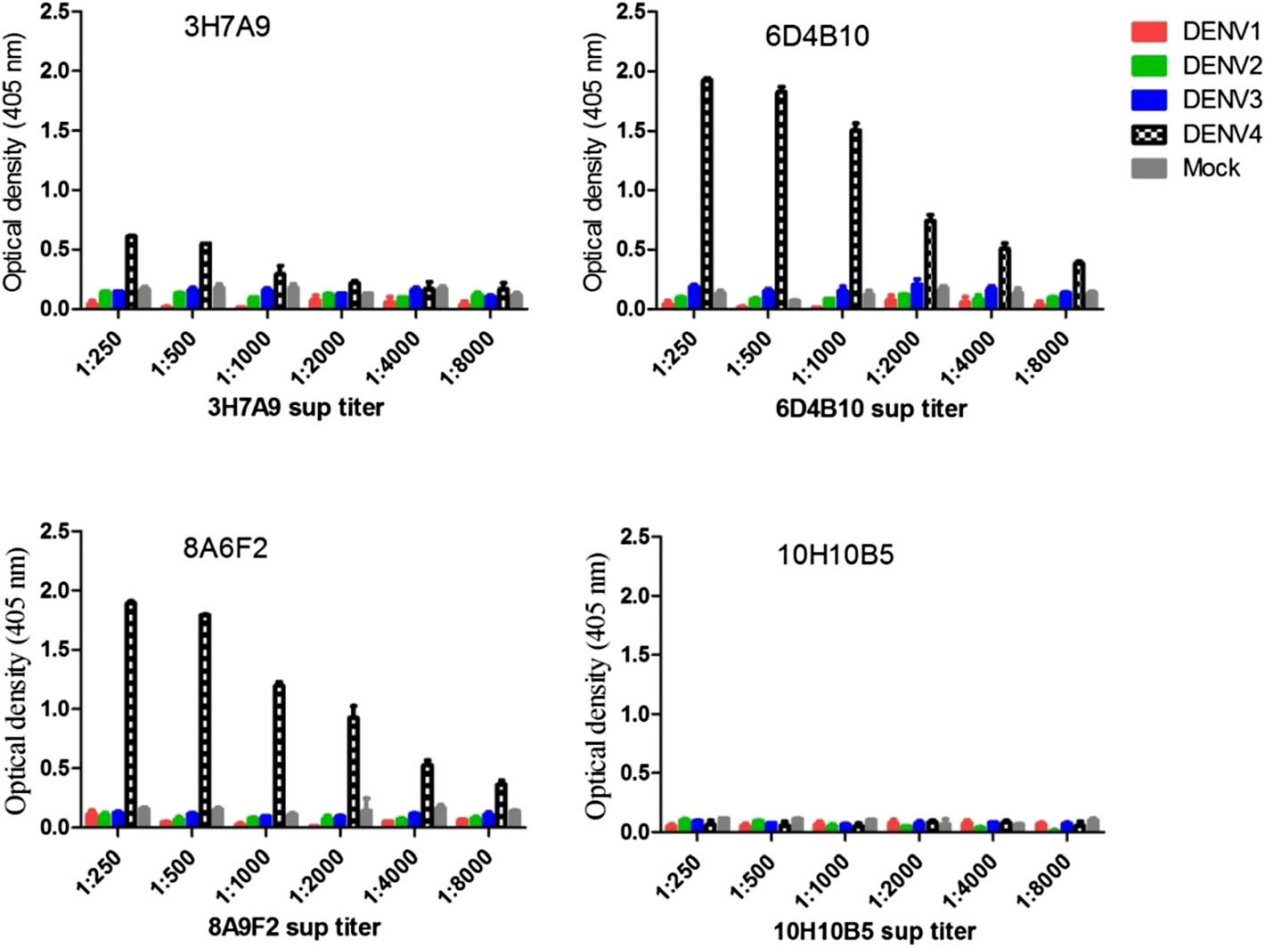
DENV-4 serotype-specificity MAbs 3H7A9, 8A6F2, 6D4B10 and 10H10B5 as determined by fixed cell ELISA. The bars showed mean optical density at 450 nm that measures MAb’s reactivity to dimeric NS1 expressed on DENV1–4-infected Vero cells. Control included mock-infected Vero cells

A total of six MAb-secreting hybridoma clones were isolated from SUMO*-DENV-4 rNS1 protein fusion primed splenocytes with Sp2/0 cells. A combination of three methods: western blot assay, iELISA and fixed cell ELISA were used to select hybridoma clones that could secrete MAbs reactive to DENV NS1. Three MAbs designated hereafter as 3H7A9, 6D4B10, and 8A6F2 had reactivity to DENV-4 rNS1 but not to SUMO* tag. Also, these three MAbs did not cross-react with any of rNS1s from the other three heterologous DENV serotypes and other related flaviviruses (yellow fever virus (YFV) and West Nile virus (WNV)). The remaining three (10H10B5, 10H8F7, 4B6C10) were found to be specific to SUMO* tag (Tables 1 and 2, Figs. 3, 4 and 5). This result was expected since the mice were immunized with SUMO*-DENV-4 rNS1 fusion protein, and the relative size of SUMO* tag, ~ 11.5 kDa. The light-chain and heavy-chain isotypes of all the six MAbs, including those which are DENV-4 serotype-specific were IgG2b and К, respectively. Competition ELISA result (data not shown) in combination with result from sequencing the three complementarity determining regions (CDR1, CDR2, and CDR3) of MAbs (Additional file 1: Figure S1) revealed that our three DENV-4 NS1 serotype-specific MAbs were distinct with different binding regions on the DENV-4 NS1.

**Table 1:** Anti-NS1 monoclonal antibodies isotype, epitope and reactivity to DENV1–4 NS1. Isotype, epitope type and iELISA results of six anti-NS1 DENV-4 monoclonal antibodies

MAb	Isotype	Epitope	iELISA (reactivity of MAbs to recombinant flavivirus NS1)							
			rNS1 DENV1	rNS1 DENV2	rNS1 DENV3	rNS1 DENV4	rNS1 WNV	rNS1 YFV	SUMO	SUMO*-NS1 fusion protein
10H8F7	IgG2b/K	Linear	–	–	–	–	–	–	+	+
4B6C10	IgG2b/K	Linear	–	–	–	–	–	–	+	+
**3H7A9**	IgG2b/K	Linear	–	–	–	+	–	–	–	+
**8A6F2**	IgG2b/K	Linear	–	–	–	+	–	–	–	+
10H10B5	IgG2b/K	Linear	–	–	–	–	–	–	+	+
**6D4B10**	IgG2b/K	Linear	–	–	–	+	–	–	–	+

*rNS1* recombinant non-structural protein 1

*DENV* dengue viruses

*SUMO* small-ubiquitin-like modifier

*YFV* yellow fever virus

*WNV* West Nile virus

Bold text are the MAbs that were reactive to rNS1 DENV-4

**Table 2:** Anti-NS1 monoclonal antibodies isotype, epitope and reactivity to DENV1–4 NS1. Monoclonal antibody reactivity to native DENV1–4 NS1 in the fixed cell ELISA and iELISA

	Reactivity to native NS1							
MAb	Fixed Cell ELISA (NS1 on surface infected Vero cells)				iELISA (NS1 in Vero cell culture supernatant)			
	DENV1	DENV2	DENV3	DENV4	DENV1	DENV2	DENV3	DENV4
10H8F7	–	–	–	–	–	–	–	–
4B6C10	–	–	–	–	–	–	–	–
3H7A9	**–**	**–**	**–**	**+**	**–**	**–**	**–**	**+**
8A6F2	–	–	–	+	–	–	–	+
10H10B5	–	–	–	–	–	–	–	–
6D4B10	–	–	–	+	–	–	–	+

*DENV* dengue viruses

### Anti-DENV-4 monoclonal antibodies were DENV-4 specific and bind to monomeric, dimeric and hexameric DENV-4 NS1 isoforms

DENV-4 serotype specificity of MAbs (3H7A9, 6D4B10 and 8A6F2) was demonstrated in fixed cell-ELISA, and in iELISA using both supernatants from DENV (1–4)-infected Vero cell culture and rNS1s of DENV1-4 (NativeAntigen, Oxfordshire, UK). (Tables 1 and 2). Western blot analysis of supernatants from the six clones after SDS-PAGE separation of SUMO*-DENV-4 rNS1 protein as well as unfused commercial DENV-4 rNS1s revealed that the binding affinity of the three MAbs (3H7A9, 6D4B10, 8A6F2) was to the linear epitopes on both monomeric and dimeric DENV-4 NS1 isoforms (Fig. 3). Additionally, these MAbs exhibited reactivity to supernatants from DENV-4 infected Vero cell culture, suggesting these 3 MAbs were reactive to hexameric DENV-4 NS1. Results from these three assays including western blot assay indicated the MAbs are capable of binding with relatively higher affinity to DENV-4 NS1 dimeric and hexameric isoforms (Tables 1 and 2 and Fig. 3).

### DENV NS1 capture ELISA was specific to DENV-4

**Fig. 6:**
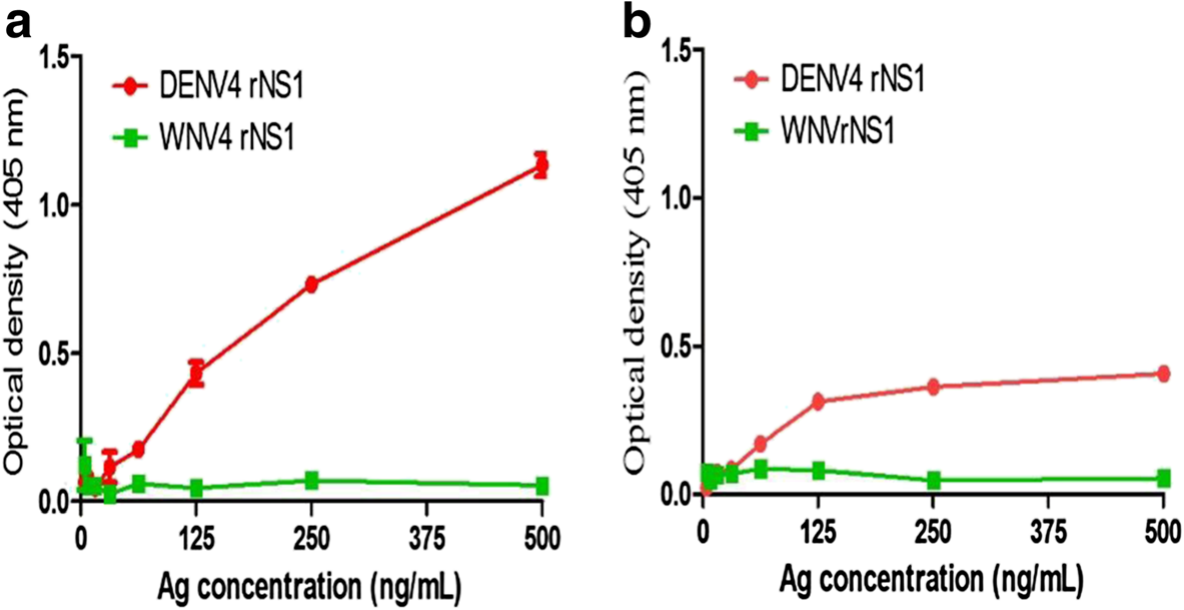
Analysis of optimal capture/detector MAb pair for development of DENV-4 serotype specific NS1 capture ELISA. The curves represent limit of detection (LOD) curve for matched pairs: **a** 8A6F2 and biotinylated 6D4B10, and **b** 6D4B10 and biotinylated 8A6F2. The optical density (OD) values at 450 nm were obtained at various concentration of DENV-4 rNS1 with an optimal concentrations for coating antibody, 5-10 μg/ml; capture antibody, 1:2000 dilution and streptavidin-tagged horseradish peroxidase (HRP-SP), 1:2000. WNV was used as the control

Results from competition ELISA (data not shown) confirmed that all the 3 MAbs recognized distinct epitopes on DENV-4 NS1, indicating a pair of these MAbs can be used for development of new NS1 detection tests specific to DENV-4 serotype. However, 3H7A9 had a relatively lower affinity (Fig. 4) and was not utilized for development of our NS1 capture assay. Thus only MAbs 6D4B10 and 8A6F2 were utilized for the development of a new NS1 capture ELISA. Based on the limit of detection (LOD) curves, shown in Fig. 6, a matched pair 8A6F2/biotinylated 6D4B10 had the lowest LOD (32.5 ng/ml) while a matched pair 6D4B10/B-8A6F2 was not robust to capture and/or detect DENV-4 rNS1 even at a higher concentration (Fig. 6b). Those matched MAbs pairs that did not perform well could be due to: (i) the denaturation/random orientation of 6D4B10 as a result of direct binding to the microtiter plate, (ii) biotin molecules might have bound on the antigen binding region of 8A6F2, or (iii) a combination of two [24, 25]. Nevertheless, result from a direct ELISA (data not shown), in which microtiter plate-wells were coated overnight with DENV-4 rNS1, and detection of bound antigen was made by biotinylated 8A6F2, suggest that the loss of antigen-antibody binding in an NS1 capture ELISA format based on match pair 6D4B10/ biotinylated 86FA2 was not linked to the biotinlyation. Determination of the best working concentration of capture and detection was done by checkerboard titration and the optimal concentrations for coating MAb, 8A6F2 and detection MAb, biotinylated 6D4B10 were found to be 10 μg/mL and 1:2000 dilution, respectively.

After optimization of the NS1 capture ELISA specific to DENV-4, we established and validated its utility using culture supernatants obtained from DENV1–4 infected Vero cells. As expected, only culture supernatants from DENV-4 infected Vero cells showed reactivity whereas culture supernatants from the other three DENV serotypes-infected Vero cell showed no reactivity.

## Discussion

In this study, we expressed SUMO*tagged DENV-4 rNS1 antigen in *Sf21* insect cells using SUMO fusion technology to produce DENV-4 specific MAbs. This baculovirus protein expression of SUMO-fused rNS1 in *Sf21* cell lines was an attractive alternative to the classic *Escherichia coli* (*E.Coli*) protein expression system [26, 27]. Our study showed that DENV NS1 protein SUMO* expression was superior to *E. Coli* and standard baculovirus antigen expression because SUMO* tagged DENV-4 rNS1 was secreted as soluble molecule in culture medium and our previous studies with *E.Coli* protein expression did not yield any immunogenic MAbs. Furthermore, the purified SUMO* tagged DENV NS1 did not require complex multi-steps for solubilizing and refolding as required by *E.Coli* protein expression. We used SUMO* as an N-terminal tag to enhance the solubility, stability and correct folding of DENV-4 rNS1 [22, 28–30]. SUMO fusion technology has previously been used for successful expression of functional hexameric DENV-2 rNS1 in insect cell lines, and the rNS1 protein product was properly glycosylated as it exist in its native form [31]. However, to the best of our knowledge, this is the first report regarding a successful expression of soluble and stable DENV-4 NS1 with a correct folding using SUMO* fusion technology. We then demonstrated the utility of purified SUMO*-DENV rNS1 for generation of MAbs that could be applied for development of immunodiagnostic tests for dengue.

Production of a properly-folded soluble NS1 protein appears to be crucial for the development of MAbs, which are reactive to epitopes on hexameric NS1. Isolation and purification of proteins expressed in *E.Coli* required solubilization in harsh detergents (e.g., SDS and urea) which denatured the target protein and required complex refolding process. DENV NS1 expressed in *E. Coli* often resulted in insoluble aggregates (i.e., inclusion bodies) [32–34] [35]. Expression of rNS1 in insect cell line such as *Sf9* and *Sf21* using a baculovirus expression system has been utilized [26, 27] but, in our experience (unpublished data), the expressed rNS1 protein also remained insoluble, and required solubilization and refolding.

In the present study, six MAb-secreting hybridomas reactive to the SUMO*-DENV-4 NS1 fusion protein were generated of which three were reactive to SUMO* tag. The remaining three MAbs (3H7A9, 6D4B10 and 8A6F2) were found to be DENV-4 serotype-specific. Serotype-specific anti-DENV NS1 MAbs is crucial for development of NS1 test that detects dengue early in the course of illness and simultaneously determines the infecting DENV serotype. Our newly developed MAbs (3H7A9, 6D4B10 and 8A6F2) are highly specific to DENV-4 as demonstrated by two different methods, fixed cell ELISA and iELISA. Of the three serotype-specific MAbs, 6D4B10 and 8A6F2 showed higher affinity to the hexameric DENV-4 rNS1 and dimeric DENV-4 NS1 expressed in Vero cells.

A competition ELISA was performed to determine if the three MAbs bind to the same or distinct epitopes of DENV-4 NS1. The results (data not shown) confirmed that all the three MAbs bind to distinct non-overlapping regions and can be used to develop sandwich immunodiagnostic assay. The distinctness of these three MAbs was further verified by sequencing the three complementarity determining regions (CDR1, CDR2, and CDR3) of MAbs. MAb 3H7A9 was DENV-4 serotype-specific but had weak affinity to DENV-4 hence was not used for assay development. Only MAbs 6D4B10 and 8A6F2 were chosen to develop an NS1 capture ELISA. In order to use biotin-streptavidin based capture ELISA format, different pairs of unbiotinylated and biotinylated MAbs were evaluated. Subsequently, a match pair of unbiotinylated 8A6F2 as a capture antibody and biotinylated (B)-6D4B10 as a detection antibody provided the most robust results compared to 6D4B10 as capture and B-8A6F2 as the detector. The reason for the poor performance of the later matched pair MAbs may be due to: (i) the denaturation/random orientation of 6D4B10 as a result of direct binding to the microtiter plate, (ii) biotin molecules binding to the antigen binding region of 8A6F2, or (iii) a combination of these two [24, 25]. Nevertheless, a direct ELISA (data not shown), in which microtiter plate-wells were coated with DENV-4 rNS1, and detected by B-8A6F2 showed reactivity of the biotinylated 8A6F2 to DENV-4 rNS1 protein, suggesting the loss of antigen-antibody binding in NS1 capture ELISA format (capture with 6D4B10 and detect with B-86FA2) was not due to the biotinlyation. This MAb pair may function efficiently if one avoids direct binding of this MAb to the microplate surface by using lower affinity capture MAb, 6D4B10 immobilization method or a linker. The newly developed DENV-4 NS1 specific capture ELISA therefore used 8A6F2 as the capture and B-6D4B10 as the detection antibody. The DENV-4 serotype-specificity was assessed using rNS1 of flaviviruses, including all four DENV serotypes and culture supernatants from Vero cell-infected with all four DENV serotypes. Because flaviviruses family of viruses may cross react in immunodiagnostic test due to their highly antigenic similarities, we included YFV and WNV to ensure DENV specificity. The results confirmed that these MAbs specifically bound to DENV-4 in the NS1 capture ELISA and not to other flaviviruses (WNV, YFV).

Another factor that could have contributed to the poor sensitivity of NS1 Ag detection tests for DENV-4 is the low level expression of NS1 in DENV-4-infected patients as compared to in dengue patients infected with the other three serotypes [5, 36]. An NS1 capture ELISA specific to DENV-4 could improve the detection of DENV-4 cases and a serotype-specific NS1 Ag test can identify dengue and the infecting DENV serotype [37]. NS1 ELISAs for each one of the four DENV serotypes have been previously described [38–40] however, none of them are commercially available and hence currently the only commonly used laboratory methods to determine DENV serotype is RT-PCR. There are two main advantages in identifying the DENV serotype: (i) risk factors for severe dengue have associated to more pathogenic DENV serotypes, and (ii) the sequence of infection of DENV serotype in primary and secondary infections is also believed to be a risk factor for severe disease. Taken together, there is a need for improvement of the current NS1 assay for detection of DENV-4 serotype. This could be done by developing multiplex lateral flow assay (LFA) that utilize broadly binding anti-DENV NS1 MAbs as well as our MAbs specific to DENV-4 NS1. However, this requires an investigation on the utility of our DENV-4 –specific MAbs for LFA.

## Conclusion

In conclusion, we generated three DENV-4-specific MAbs directed against SUMO*-DENV-4 rNS1 fusion protein. Based on our characterization results and competition ELISA, the NS1 capture ELISA for potential early detection DENV-4 infection was established using a combination of two highly sensitive MAbs: 8A6F2 as coating antibody, biotinylated 6D4B10 as detection antibody and an optimized protocol was developed. The assay was specific to DENV-4 with no cross-reactivity to other three DENV serotypes and other heterologous flaviviruses. Our preliminary results indicated that our MAbs are useful reagents for development immunodiagnostic assays (e.g., ELISAs and LFA) that specifically detect DENV-4 infection. These DENV serotype specific MAbs have the potential to change the way we detect dengue by providing tests that are user friendly to resource poor regions to adopt them for diagnosing dengue. Given that the current commercial NS1 tests are less sensitivity to DENV-4, our paired MAbs could be useful for development of multiplex assay that detects all four serotypes simultaneously in regions where DENV-4 circulates. However, one major limitation of this study is that these MAbs were not tested using DENV-4 clinical specimens thus these MAbs require further validation. Furthermore a full validation testing should be conducted to better understand the limitations of these MAbs in flavivirus clinical specimens.

## Additional files

Additional file 1: Figure S1. The alignment of the sequences of the three complimentary determining regions (CDR1, CDR2, CDR3) of monoclonal antibodies 3H7A9, 8A6F2 and 6D4B10 showing they are distinct. (DOCX 14 kb)
