# Supplementary material for: Development and characterization of serotype-specific monoclonal antibodies against the dengue virus-4 (DENV-4) non-structural protein (NS1)
Source: Virol J. 2018 Feb 6;15:30. doi: 10.1186/s12985-018-0925-7 (PMC5801815; doi:10.1186/s12985-018-0925-7)
Supplement: Supplementary file 1 — The alignment of the sequences of the three complimentary determining regions (CDR1, CDR2, CDR3) of monoclonal antibodies 3H7A9, 8A6F2 and 6D4B10 showing they are distinct. (DOCX 14 kb) [file 12985_2018_925_MOESM1_ESM.docx]

CLUSTAL O(1.2.4) multiple sequence alignment

**Multiple alignment of MAbs’ Heavy chain Amino acid sequence: Leader sequence-FR1-CDR1-FR2-CDR2-FR3-CDR3-FR4**

8A6F2 -MRVLILLCLFTAFPGILSDVQLQESGPDLVKPSQSLSLTCTVTGYSITSGYNWHWIRQF

6D4B10 MEWSWIFLFLLSGTAGVHSEVQLQQSGPELVKPGASVKMSCKASGYTFTS-YVMHWVKQK

3H7A9 MKCSWVIFFLMAVVTGVNSEVQLQQSGAELVKPGASVKLSCTASGFNIKD-TYMHWVKQR

::: *:: *: *:****:** :****. *:.::*..:*:.:.. **::*

8A6F2h PGNKLEWMGYIH-YSGGTNYNPSLKSRISITRDTSKNQFFLQLNSVTTEDTATYYCARRT

6D4B10h PGQGLEWIGYLNPYNDDTKYNEKFKGKATLTSDKSSSTAYMELSSLTSEDSAVYYCAYGP

3H7A9h PEQGLEWIGRIDPANGNTQYGPKFQGKATITADTSSNTAYLQLSSLTSEDTAVYYCARGG

* : ***:* :. ...*:*. .::.: ::* *.*.. :::*.*:*:**:*.****

8A6F2h G--------TVPFAYWGQGTLVTVSA

6D4B10h ---------PYALDYWGQGTSVTVSS

3H7A9h IFYYYGSSYSYAMDYWGQGTSVTVSS

: ****** ****:

**Multiple alignment of MAbs’ Light chain Amino acid sequence: Leader sequence-FR1-CDR1-FR2-CDR2-FR3-CDR3-FR4**

3H7A9L MMSSAQFLGLLLL--CFQGTRCDIQMTQTTSSLSASLGDRVTISCRASQDI-SNYLNWYQ

6D4B10L MDFQVQIFSFLLISASVIMSRGQIVLTQSPAIMTASLGERVTMTCTASSSVSSSYLHWYQ

8A6F2L MDFHVQIFSFMLISVTVILSSGEIVLTQSPALMAASPGEKVTITCSVSSSISSSNLHWYQ

* .*::.::*: . : :* :**: : ::** *::**::* .*..: *. *:***

3H7A9L QKPDGTVTLLIYYTSRLHSGVPSRFSGSGSGTDYSLTISNLEQEDIATYFCQQGNTLPRT

6D4B10L QKPGSSPKLWIYSTSNLASGVPARFSGSGSGTSYSLTISSMEAEDAATYYCHQYHRSPYT

8A6F2L QKSETSPKPWIYGTSNLASGVPVRFSGSGSGTSYSLTISSMEAEDAATYYCQQWSSYPLT

** : . ** **.* **** *********.******.:* ** ***:*:* * *

3H7A9L FGGGTKLEIK

6D4B10L FGGGTKLEIK

8A6F2L FGGGTKLEIK

**********
